# Determination of pararosaniline hydrochloride in workplace air

**DOI:** 10.1007/s10661-019-7568-z

**Published:** 2019-06-17

**Authors:** J. Kowalska, A. Jeżewska

**Affiliations:** Department of Chemical, Aerosol and Biological Hazards, Central Institute for Labour Protection – National Research Institute, Czerniakowska 16, 00-701 Warsaw, Poland

**Keywords:** 4,4′-(4-iminocyclohexa-2,5-dienylidenemethylene)dianiline hydrochloride, Basic Red 9 monohydrochloride, Pararosaniline hydrochloride, Diamant fuchsine, Carcinogens, Occupational exposure, Analysis of air, Chromatography, Validation

## Abstract

Pararosaniline hydrochloride (CPR) is a dye used for colouring paper, leather and natural and artificial fibres. It is also used in analytical and microbiological laboratories. It is a carcinogenic substance of category 1B. In analytical chemistry, it is used for detecting the following among others: bromates, formaldehyde, ozone, sulphite and sulfur dioxide. CPR is a dye commonly used in microbiology for staining preparations, for staining bacteria, antibodies or other organisms. In Poland, about 800 employees were exposed to this substance. The lack of methods for the determination of pararosaniline hydrochloride in workplace air makes it impossible to assess the occupational exposure of workers to this substance. For this reason, a determination method has been developed, which allows for the determination of pararosaniline hydrochloride in the air. This method makes it possible to determine the concentration of CPR in the air at the workplace within the range from 0.002 to 0.04 mg/m^3^ (for an air sample of 120 L). The method is based on the adsorption of pararosaniline hydrochloride present in the workplace air on a polypropylene filter, eluting the substance deposited on the filter with methanol and analysing the solution thus obtained using high-performance liquid chromatography with a diode array detector (wavelength of 544 nm). Using an Ultra C18 (250 mm length) chromatographic column at a temperature of 23 °C and the mobile phase of methanol:0.1% phosphoric acid(V) (95:5, *v*/*v*) at flow rate of 0.6 mL/min makes it possible to determine the content of pararosaniline hydrochloride in the presence of aniline, nitrobenzene and 4-tolylamine. Limit of detection and limit of quantification were 0.17 ng/mL and 0.51 ng/mL, respectively.

## Introduction

Pararosaniline hydrochloride (CPR; CAS No. 569-61-9) also known as parafuchsine hydrochloride, C.I. basic red monohydrochloride, C.I. 42500, magenta 0, 4,4′-(4-iminocyclohexa-2,5-dienylidenemethylene)dianiline hydrochloride or Basic Red 9 is a solid with a red colour in a green metallic sheen. It is slightly soluble in water and ether and soluble in ethanol, methanol, benzyl alcohol and ethylene glycol methyl ether (Duman et al. 2015; IARC 2012; HSDB 2018).

CPR belongs to the triarylmethane class dyes and is used for colouring various types of materials and products: paper, leather, textiles, glass, waxes and cosmetics as well as for the manufacture of inks and paints (Martins et al. 2006; Zargar et al. 2009; IARC 2010; Perdih and Perdih 2011; Nadaroglu et al. 2015; HSDB 2018).

In analytical chemistry, it is used for determining the following among others: bromates, formaldehyde, ozone, sulphite and sulfur dioxide (Miksch et al. 1981; Dasgupta 1981; Steiner et al. 1987; Romele 1998; Goyal 2001; Segundo and Rangel 2001; González-Rodríguez et al. 2002; McFeeters and Barish 2003; Simkhada et al. 2005; Li and Zhao 2006; Goyal 2006; Ćwikła-Bundyra 2012; Pundir and Rawal 2013; Almeida Jr et al. 2018).

CPR is a dye used in microbiology for staining preparations, inter alia, for staining bacteria, antibodies or other organisms in different types of tissues (Schrijver et al. 2000; Kiernan 2004). It is used to prepare the Schiff reagent required for the staining procedure of cytological and histological material (Lyon et al. 2002).

CPR is classified as a carcinogenic substance of category 1B (Carc. 1B) with an assigned hazard statement H350: May cause cancer (EC No. 1272/ 2008) because it may cause bladder cancer (Case and Pearson 1954; Piolatto et al. 1991; Bennett and Davis 2002; Pira et al. 2010; IARC 1993, 2010, 2012).

The routes of potential human exposure to CPR are dermal contact, inhalation and ingestion. Laboratory personnel who use and handle basic fuchsin dye might be exposed to Basic Red 9 monohydrochloride (HSDB 2018).

From the resources of the Central Register of Data on Exposure to Carcinogenic or Mutagenic Substances, Mixtures, Agents or Technological Processes, maintained by the Nofer Institute of Occupational Medicine, Łódź, Poland, it transpires that in Poland in 2016, 800 employees (including 702 women) in 98 enterprises were occupationally exposed to CPR. They were mainly employees of factory, healthcare, environmental protection and veterinary laboratories as well as universities, scientific institutes and others.

The value of maximum admissible concentration (MAC) for this substance in workplace air is not specified in Poland.

In the literature, there are no methods of pararosaniline hydrochloride determination in the workplace air, and only methods for determining CPR content in an aqueous solution (Zargar et al. 2009; Tokalioglu et al. 2015), in industrial wastewater (Moawed and Alqarni 2013), in textiles and in toys (Ma et al. 2010; Huang et al. 2005; Guo et al. 2018) are available. Its poor biodegradability and carcinogenicity properties were reasons for the research conducted into the disposal of pararosaniline hydrochloride from the environment, mainly from wastewater (Martins et al. 2006; Gupta et al. 2008; Duman et al. 2015; Torun and Şolpan 2015; Nadaroglu et al. 2015; Sivarajasekar et al. 2016; El Haddad 2018; Ning et al. 2018; Li et al. 2018; Zhou et al. 2018).

Different separation and detection methods are employed for the determination of CPR among other dyes. Arráez Román et al. (2005) applied the interfacing capillary electrophoresis and surface-enhanced resonance Raman spectroscopy for this purpose.

Ding et al. (2009) optimised liquid chromatography coupling with electrospray ionisation tandem mass spectrometry for simultaneous separation and identification of nine carcinogenic dyes (inter alia Basic Red 9) prohibited in textile materials.

The spectrophotometric method is most commonly used for the determination of pararosaniline hydrochloride (Martins et al. 2006; Gupta et al. 2008; Zargar et al. 2009; Moawed and Alqarni 2013; Duman et al. 2015; Tokalioglu et al. 2015; Zhou et al. 2018; El Haddad 2018). The optical absorption spectrum of pararosaniline hydrochloride showed a maximum absorption band at 546 nm for the aqueous solution (Guha and Mohan 2004; Lyon et al. 2002; Martins et al. 2006; Duman et al. 2015) and 560 nm in solutions of 1-butanol (Vinitha et al. 2007). Therefore, in order to calculate the concentrations of CPR in solutions, the absorbance is determined at an absorption wavelength of 540–554 nm.

European standards recommend the determination of carcinogenic and allergenic dyes in dyed, printed or coated textile products (PN-EN ISO 16373-3 2014) and toys (PN-EN 71-11 2007) carried out by liquid chromatography coupled with UV/Vis spectrophotometric detection and mass spectrometry.

The standard PN-EN 16373-3 ( 2014) makes it possible to identify some carcinogenic dyestuffs in dyed, printed or coated textile goods. The standard describes analytical techniques applied to determine the content of eleven carcinogenic dyes: high-performance liquid chromatography-photodiode array detector (HPLC-DAD) and high-performance liquid chromatography-mass spectrometer (HPLC-MSD). Dyestuffs, isolated from a sample of textile material (using triethylamine/methanol) in a multi-stage process, were dissolved in methanol finally. Quantification is executed by the method of HPLC-DAD. It is recommended that dyes be separated on Inertsil ODS-3 (150 × 3.0 mm; 5 μm) column at a temperature of 45 °C and with the gradient programmed mobile phase consisting of acetonitrile and ammonium acetate (10 mMol/L). The measuring can be conducted at the analytical wavelengths of 350, 480, 500, 510, 540 (for Basic Red 9) and 600 nm. The concentration range of calibration solutions of dyes in methanol is from 1 to 100 μg/L. When a large number of foreign substances are detected in extract, HPLC-MSD is recommended for identification and quantification.

An analytical method based on HPLC combined with ultraviolet-visible spectrometric detection (UV/Vis) and tandem mass spectrometry (MS-MS) has also been used by Ma et al. (2010) for the determination of sixteen carcinogenic and allergenic dyestuffs in toys. The separation was performed with Zorbax Extend C18 column (150 mm × 2.1 mm; 5 μm) at a temperature of 25 °C with a mobile phase of acetonitrile-tetrahydrofuran-citrate buffered tetrabutylammonium hydroxide mixture under gradient elution. Each dyestuff was detected and quantified at its respective maximum ultraviolet-visible absorption wavelength for optimum sensitivity (Basic Red 9; 540 nm).

The presence of carcinogens in work environments poses a big problem for employers, not to mention the personnel exposed to them. The carcinogen and mutagen directive (Directive 2004/37/EC 2004) sets out the minimum standards for the protection of workers from occupational carcinogens. Employers’ duties include the following: identifying carcinogenic and mutagenic substances and mixtures, assessing the risk to workers and regular monitoring of workers’ exposure to determine any health risk and deciding the measures to be taken.

Screening measurements of the concentration of carcinogens are performed to obtain quantitative information on exposure levels. Such information shall be useful in identifying potential health risks and in assessing health risks.

Due to the occurrence of pararosaniline hydrochloride in the working environment in Poland and the classification of this substance as carcinogenic of category 1B (Carc. 1B), it has become necessary to determine its concentrations in the workplace air at a sufficiently low level.

The aim of the research was to develop a method of collecting air samples at a workplace enabling sampling using an individual dosimetry method, where the sampler is placed in the worker’s breathing zone, selecting the appropriate filter on which the test substance is retained, and a solvent, which quantitatively elutes the substance from the filter. The developed quantitative method will make it possible to determine the concentration of carcinogenic CPR in the workplace air, which in turn will allow the determination of exposure indicators and facilitate the assessment of occupational risks for workers.

## Materials and methods

### Equipment

The tests were carried out using a liquid chromatograph by Agilent Technologies (Waldbronn, Germany), series 1200, with a diode array detector (DAD) coupled online. Samples were introduced using the autosampler G2258-90010 (Agilent Technologies). ChemStation software was used to control the process of determining and collecting data. For separating the analytes, an Ultra C18 chromatography column (250 × 4.6 mm) with *d*_p_ = 5 μm, with a pre-column (10 × 4.0 mm) (Restek, Bellefonte, PA, USA), was used. A Gilair 5 Aspirator (Sensidyne, Clearwater, FL, USA) for personal air sampling and a WL-2000 mechanical shaker (JWElectronic, Warsaw, Poland) to carry out CPR recovery from the filter were both applied.

### Material and reagent

The following reagents were used in the tests: pararosaniline hydrochloride (CPR) from Merck (Darmstadt, Germany), aniline, nitrobenzene (Sigma-Aldrich, Steinheim, Germany), methanol (J. T. Baker, Deventer, the Netherlands), phosphoric acid(V), 4-tolylamine (POCh, Gliwice, Poland) and high-purity water obtained from the Milli-Q apparatus (Millipore, Bedford, MA, USA). Reagents of HPLC grade were used during the tests.

Materials used were the following: glass fibre filters, GF/A with a diameter of 25 mm by Whatman (Maidstone, UK), filters made of poly(propylene), FIPRO with a diameter of 25 mm (Textile Research Institute, Łódź, Poland), poly(tetrafluoroethylene) filters, PTFE with a diameter of 25 mm (SKC, Pittsburgh, PA, USA), poly(vinyl chloride) filters and PVC with a diameter of 25 mm (SKC, Pittsburgh, PA, USA).

### Methodology

#### Sample preparation

Air samples containing CPR (120 L) were collected on filters. Two millilitres of methanol was used for recovery of CPR deposited on the filter, and the contents were shaken for 30 min. The solution from above the filter was then determined by means of chromatography using an Ultra C18 column for HPLC with a pre-column.

#### Chromatographic conditions

**Fig. 1:**
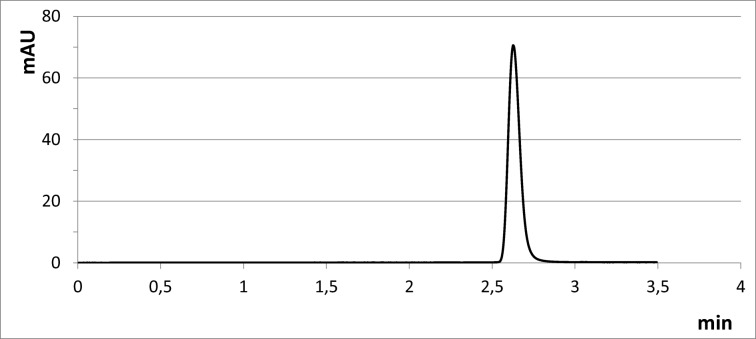
Chromatogram of the CPR standard solution in methanol

The temperature of the Ultra C18 column was 23 °C. The mobile phase flow rate was 0.6 mL/min. As the mobile phase, methanol:0.1% phosphoric acid(V) (95:5, *v*/*v*) was used. The volume of the sample to be dispensed was 10 μL. A DAD detector was used for detection. The analytical wavelength *λ* of the DAD detector was 544 nm. These conditions enabled the determination of pararosaniline hydrochloride in the presence of aniline, nitrobenzene and 4-tolylamine, i.e. substances that may co-exist with CPR in the work environment. These substances do not interfere with the CPR determination at the analytical wavelength of 544 nm (Fig. 1).

#### Examination of recovery

In order to determine the degree of CPR recovery from the FIPRO filter, the following activities were carried out: 0.24 μg of pararosaniline hydrochloride (dosing 10 μL of a CPR solution in methanol at 24 μg/mL), as well as 2.4 μg and 4.8 μg of CPR, was applied onto filters (6 pieces each) by subsequent dosing of 10 μL and 20 μL of a CPR solution in methanol at 240 μg/mL. The filters were then dried and 120 L of air was passed through them. CPR recovery was carried out with methanol (2 mL), shaking the contents for 30 min. Solutions from above the filters were determined by means of chromatography. Pararosaniline hydrochloride determination was also carried out in comparative solutions made in the same way but without a filter. A method is considered accurate if the average recovery obtained from a series of measurements is between 97 and 103%.

#### Calibration and precision

Calibration assays were performed for six CPR standard solutions in methanol in the concentration range from 0.12 to 2.4 μg/mL. Three series of calibration solutions were made, which were subjected to chromatographic analysis. Ten microlitres of standard solutions with increasing concentrations was introduced into the chromatograph. The analytical precision of the calibration determinations was evaluated on the basis of the analysis of three series of eight working solutions with CPR concentrations successively at 0.12 (first series), 1.2 (second series) and 2.4 μg/mL (third series). For the following analysis, each of three series of standard solutions, standard deviations and the coefficients of variation was calculated.

#### Method validation

Validation parameters were determined in accordance with the requirements contained in the European standard PN-EN 482 ( 2016). The overall approach, including both sampling and analytical methods, was tested by determining the recovery, linearity, sensitivity, precision and expanded uncertainty. The limit of detection and the limit of quantification are based on the results of the analysis of ten independent peak area measurements with the CPR retention time for three independently prepared blank samples. To calculate the limit of detection (LOD), the following dependence was used:

1 $$ \mathrm{LOD}=\frac{3.3\cdot {s}_o}{b} $$

where *b* is the coefficient of the directional calibration line (calibration coefficient) obtained when determining linearity, and *s*_*o*_ is the standard deviation of results obtained for a series of blank samples.

The LOQ value was calculated as a multiple of the determined LOD value: LOQ = 3 LOD.

The total analytical precision (*V*_*c*_) which includes laboratory precision for the measurement range and sampling method was determined from the following formula:

2 $$ {V}_c=\sqrt{V_z^2+{V}_p^2} $$

where *V*_*p*_ is the precision of the sampling device (*V*_*p*_ = ± 5%), and *V*_*z*_ is the average precision of three levels within the range, calculated from the formula:

3 $$ {V}_z=\sqrt{\frac{\sum \left({n}_j-1\right)\cdot {V}_i^2}{\sum \left({n}_j-1\right)}} $$

where *n*_*j*_ is the number of repeated samples (*n*_*j*_ = 8), and *V*_*i*_ is the variation coefficient for the concentration level.

The total analytical precision value (*V*_*c*_) is taken into account in the calculation of combined standard uncertainty. The expanded uncertainty is obtained by multiplying the combined standard uncertainty with a coverage factor of 2 (*p* = 0.95).

## Results and discussion

The tests were carried out to determine the conditions for collecting air samples containing pararosaniline hydrochloride to ensure the quantitative separation of substances from the workplace air. Typically, the particles, which have been collected into a filter, an adsorbent, or a similar system, are extracted with a suitable solvent and subsequently analysed. Pararosaniline hydrochloride is a solid under normal conditions; therefore, filters must be used to isolate it from the air. The following filters were used for the tests: PVC, PTFE, GF/A and FIPRO, which can be easily placed in the worker’s breathing zone as per the principles of individual dosimetry (PN-Z-04-008-7). Using the individual dosimetry method, the most reliable results of occupational exposure assessment are obtained.

### Preliminary investigations of CPR recovery from filters

Initial investigations of the degree of CPR recovery from the filters (PVC, PTFE, FIPRO and GF/A) were tested as follows: 100 μL of a CPR solution in methanol at 24 μg/mL was applied onto filters (3 pieces each) and allowed to dry. The dried filters were extracted with 2 mL of methanol. After shaking (30 min), the solutions from above the filter were analysed chromatographically under the conditions given above. Comparable values of recovery were obtained for all the tested filters (about 98%) (Table 1). Due to the lower cost of the GF/A and FIPRO filters in comparison with the others, these filters were chosen for further research.

**Table 1:** Comparison of filters (CPR recovery from filters with methanol). Ultra C18 column, DAD detector (*λ* = 544 nm)

Type of filter	Average area of CPR peaks from recovery solutions	Average area of peaks from comparative solutions	Recovery (%)	Average recovery (%)
PVC	329.9	335.8	98	98
	330.2		98	
	331.15		99	
PTFE	327.7		98	97
	330.1		98	
	322.7		96	
FIPRO	323.5		96	98
	330.2		98	
	329.65		98	
GF/A	328.0		98	98
	327.3		97	
	329.65		98	

### Investigation of air sampling conditions

In order to determine the conditions of air sampling and the selection of one type of filter, a system consisting of two GF/A filters connected in a series and a suction pump with a constant volume of air flow controlled by a rotameter and an identical set consisting of two FIPRO filters were assembled. In each set, the first filter contained the test substance (12 μg of substance) and the second filter was clean, i.e. the so-called control one. One hundred twenty litres of air was passed through the system with a constant flow rate of 120 L/h, 60 L/h and 20 L/h; after which, the filters were left in the desiccator until the following day. The solution obtained after the extraction of CPR from the filters (2 mL of methanol) was analysed by means of chromatography. The results are presented in Table 2. The results obtained indicate the retention of CPR on the first filter. The recovery of substances from the FIPRO filters was higher than that from the GF/A filters. For this reason, the FIPRO filters were selected for further investigations, with just one filter being sufficient, as the investigated substance does not pass to the second filter. On the basis of the results obtained, the method of collecting air samples containing CPR was determined: 120 L of air being tested is passed through a polypropylene filter with a volume stream not higher than 120 L/h. Such a procedure allows for collecting one air sample over 6 h in the worker’s breathing zone, in accordance with the principles of individual dosimetry included in the Polish standard PN-Z-04008-7 (2002).

**Table 2:** Exemplary CPR adsorption studies on FIPRO and GF/A filters. Ultra C18 column, DAD detector (*λ* = 544 nm)

Type of filter	Flowrate of air (L/h)	Area of CPR peaks in recovery solutions	
		1st filter	2nd filter
FIPRO	0	1,830	–
FIPRO	120	1,750	–
FIPRO	60	1,722	–
FIPRO	20	1,735	–
GF/A	0	1,508	–
GF/A	120	1,476	–
GF/A	60	1,308	–
GF/A	20	1,240	–

### Examination of recovery

The recovery was calculated by comparing the analytical results for the extracted samples in three concentrations with the non-extracted standards (assumed to be 100% recovery). Recovery of the analyte should be between 95 and 105%. The results are presented in Table 3. The average recovery rate was 98%. The relative standard deviation was < 10% for all values.

**Table 3:** Determination of CPR recovery rate from the FIPRO filter. Ultra C18 column, DAD detector (*λ* = 544 nm)

CPR mass applied onto the filter (μg)	Average area of peaks in recovery solutions	Average area of peaks in comparative solutions	Relative standard deviation (%)	Average recovery (%)
0.24	32.2	33.0	2.2	98
2.4	323.0	330.6	0.36	98
4.8	653.9	664.0	0.57	98

### Calibration and precision

The calibration curve consisted of six calibration points using replicated samples. The area peak of CPR vs. the concentration was plotted and produced a linear curve for the concentration range used (0.12–2.2 μg/mL) with a correlation coefficient of 1. A typical equation for the calibration curve was *y* = 278*x* + 0.13 (Table 4) where *y* represents the peak area of CPR and *x* represents the CPR concentration in microgrammes per millilitre.

**Table 4:** Calibration parameters for the three measurement series

Parameter	Measuring series		
	I	II	III
Calibration curve (*y* = *bx* + *a*)	*y* = 279*x* − 0.23	*y* = 277*x* + 0.14	*y* = 277*x* + 0.50
Correlation coefficient, *r*	1	1	1
Average value of the calibration coefficient	278		
Coefficient of variation of the calibration coefficient (%)	0.49		

The precision of the calibration determinations was evaluated on the basis of the analysis of three series (0.12, 1.2 and 2.4 μg/mL) of eight CPR standard solutions in methanol. The coefficients of variation for successive concentration levels are 1.79, 0.41 and 0.74%, respectively. The total analytical precision determined (according to formula 2) was 5.13%.

### Validation results

Validation parameters were determined in accordance with good laboratory practise and the requirements contained in the European standard PN-EN 482 (2016). The validation data obtained on the basis of the results of the tests performed are presented in Table 5 and are in agreement with EN 482 requirements which are specific for working environment. The limit of quantification was 0.51 ng/mL. This corresponds to 0.001 μg per sample. The relative quantification limit was 8.5·10^−6^ mg/m^3^ for an air sample volume of 120 L. The expanded uncertainty for those methods was acceptable and is equal to 23%.

**Table 5:** Validation data for the determination method of pararosaniline hydrochloride

Parameter	Value
Measuring range	0.002 ÷ 0.04 mg/m^3^
Amount of air collected	120 L
Lineal concentration range	0.12 ÷ 2.4 μg/mL
Limit of detection (LOD)	0.17 ng/mL
Limit of quantification (LOQ)	0.51 ng/mL
Overall precision of the test	5.1%
Expanded uncertainty	23%

## Conclusion

As a result of the research conducted, a method for the determination of pararosaniline hydrochloride in the workplace air using high-performance liquid chromatography with a diode array detector was developed. The method involves passing the tested air containing pararosaniline hydrochloride through a polypropylene filter, extracting the substance from the filter with methanol and chromatographic analysis of thus obtained solution using the Ultra C18 column (250 × 4.6 mm; 5 μm). The method was validated in accordance with the European standard PN-EN 482. Good validation results were obtained. The developed method enables the determination of pararosaniline hydrochloride in the air of the work environment in the concentration range of 0.002 to 0.04 mg/m^3^ and can be used to assess occupational exposure to this substance.
